# Immunological Manifestations of Hepatitis E-Associated Acute and Chronic Liver Failure and Its Regulatory Mechanisms

**DOI:** 10.3389/fmed.2021.725993

**Published:** 2021-08-09

**Authors:** Jian Wu, Bai Ling, Naizhou Guo, Guanghua Zhai, Meifen Li, Yurong Guo

**Affiliations:** ^1^Department of Clinical Laboratory, Gusu School, Suzhou Municipal Hospital, The Affiliated Suzhou Hospital of Nanjing Medical University, Nanjing Medical University, Suzhou, China; ^2^Department of Pharmacy, The First People's Hospital of Yancheng City, The Yancheng Clinical College of Xuzhou Medical University, Yancheng, China; ^3^Department of Clinical Laboratory, The First People's Hospital of Yancheng City, The Yancheng Clinical College of Xuzhou Medical University, Yancheng, China; ^4^Department of Laboratory Medicine, Yancheng Hospital of Traditional Chinese Medicine, Affiliated to Nanjing University of Traditional Chinese Medicine, Yancheng, China

**Keywords:** hepatitis E virus, acute and chronic liver failure, immune manifestations, mechanism, microenvironment

## Abstract

Hepatitis E virus (HEV) is a common cause of viral hepatitis in developing countries, most commonly transmitted through the fecal-oral route. The virus is mainly of genotypes (GT) 1 and GT2 genotypes, and patients usually show symptoms of acute hepatitis. Due to the rising trend of HEV serological prevalence in global population, HEV has become an important public health problem in developed countries. Severe hepatitis caused by HEV includes acute and chronic liver failure (ACLF). ACLF frequently occurs in developed countries and is caused by overlapping chronic liver diseases of HEV with genotypes GT3 and GT4. Because the onset of hepatitis E is closely associated with immunity, it is critical to understand the immunological mechanism of hepatitis E associated with acute and chronic liver failure (HEV-ACLF). This review discusses the immunological manifestations and mechanisms of HEV-ACLF, intrahepatic immune microenvironment and treatment, and raises outstanding questions about the immunological mechanism and treatment of the disease.

## Introduction

Hepatitis E is a liver infection caused by the single-stranded RNA hepatitis E virus (HEV) and usually a self-limited disease (1). However, the disease may develop into severe hepatitis in patients with altered immune responses, such as pregnant women. Recent studies have reported that HEV infection can lead to chronic hepatitis in patients with low immunity, such as HIV infected patients, organ transplant recipients and patients suffering from malignant diseases (2–5). Currently, there are four well-characterized HEV genotypes that can infect mammals, among them, genotypes (GT) 1 and 2 cause human infection, while genotypes 3 and 4 bring about zoonotic disease (6) that can infect a wide range of hosts. HEV is mainly transmitted by the fecal-oral route. Usually, an immune-capable individual can eliminate the virus spontaneously without causing complications. However, in some cases where the immune system is unable to resist the virus, HEV infected patients will experience symptoms of acute viral hepatitis such as jaundice, hepatomegaly, vomiting, nausea, abdominal pain and fever (7). In patients with low immunity, HEV virus can cause chronic liver infection, liver failure and extrahepatic symptoms. A large number of studies have shown that the immune response, rather than the virus itself, is the key factor driving the occurrence of hepatitis E (6). As a self-limited disease, the treatment principle is supporting treatment in patients with normal immune function, while for cancer patients and patients who receive long-term immunosuppressive therapy after organ transplantation, antiviral therapy and temporary suspension of immunotherapy can alleviate the symptoms of some patients (8). HEV infection can cause acute decompensation of chronic liver disease, leading to liver failure and death of patients, known as acute chronic liver failure (ACLF). The Asia-Pacific Association for the Study of the Liver (APASL) defines ACLF as acute liver impairment characterized by jaundice and coagulation disorders, complicated by ascites or encephalopathy within 4 weeks in patients with previously diagnosed or undiagnosed chronic liver disease. ACLF is defined by the American Society of Liver Diseases as an acute worsening of preexisting chronic liver disease, usually associated with an emergency event and associated with increased mortality from multi-system and organ failure at 3 months (9). Both definitions refer to the basic characteristics of acute liver damage or acute exacerbation of chronic liver disease in patients. The superposition of HEV infection has been shown to be an important cause of liver injury in patients with chronic liver disease, who also have a higher mortality rate than patients with stable compensatory cirrhosis. Therefore, ACLF is characterized by acute deterioration of compensatory chronic liver disease in patients with stable cirrhosis, and the main clinical manifestations are hyperbilirubinemia (with clinical jaundice), hepatic encephalopathy, and decreased liver function, leading to hypoproteinemia and shortened prothrombin time (10). The genotypes of HEV causing acute and chronic liver failure in chronic liver disease are GT3 and GT4, which are different from those caused by GT1 and GT2 HEV during pregnancy. ACLF and ALF present similar clinical symptoms and signs in the acute phase and are difficult to distinguish from each other. However, the prognosis of ACLF is worse, and the mortality is also significantly higher than that of ALF (11). The early mortality of ACLF is as high as 50–90% (12–14). In recent years, a large number of clinical studies have shown that immune response plays a very important role in the occurrence and development of ACLF, but the exact pathogenesis of ACLF remains to be clarified.

In this paper, the manifestations of immune response and the regulatory mechanisms in the occurrence and development of hepatitis E associated acute and chronic liver failure (HEV-ACLF) were elaborated, and the mechanisms underlying severe hepatitis E were summarized and explored. This article will provide a new idea for clinical treatment and research of hepatitis E.

## Immunological Manifestations and Mechanisms of Acute Hepatitis E (AHE)

HEV usually infects susceptible populations through the fecal-oral route, and the clinical manifestations of patients vary greatly, from asymptomatic infection to uncomplicated acute viral hepatitis and severe fulminant liver failure. The reasons for the various degrees of disease severity and the pathogenesis of the disease remain unclear. However, more and more studies have found that the virus is not the driving factor of liver injury, and the main cause of liver tissue injury in AHE patients may be the host immunity (6).

Under normal conditions, the symptoms of acute hepatitis are presented after HEV infection, which are mainly manifested by the rapid increase of serum liver enzymes such as alanine aminotransferase (ALT), aspartate aminotransferase (AST), γ-glutamyl transpeptidase (GGT), alkaline phosphatase (ALP), as well as jaundice. Several studies in developing countries such as India have shown the occurrence of toxemia in AHE patients. Reverse transcription-polymerase chain reaction (RT-PCR) was used to detect HEV RNA in serum and feces during the onset of the disease, and the results showed that the positive rate of serum HEV RNA in the 1st week of onset was about 86.7 and 6.6% in the 6th week of the disease. Meanwhile, the excretion of HEV RNA in the stool was about 70% in the 1st week. In the 4th week, it was reduced to 20%, and it turned negative around the 5th week (15). Approximately 80% of patients have detectable anti-HEV immunoglobulin IgM, and the HEV IgM appears 1 week after the onset of symptoms. In the following 6 weeks, IgM-positive cases showed a monotonic decline. It decreased to 18.3% in the 7th week (16). Based on the statistics of the above detection indicators, anti-HEV IgM, serum HEV RNA and fecal virus disappear in a few weeks, and fecal virus disappeared first, followed by HEV RNA, and then anti-HEV IgM. The longest duration of HEV viremia was 42 days, that of HEV fecal excretion was 28 days, and IgM was 49 days (15). Several studies have found that the fecal excretion rate of the viruses is significantly lower than the detection rate of serum HEV RNA. Therefore, fecal virus shedding is not an accurate and desirable diagnostic method. The positive rate of HEV RNA was higher than that of anti-HEV IgM, suggesting that detection of HEV RNA may be a good indicator of persistent HEV infection and diagnosis (15).

A study of 60 AHE patients in a tertiary care facility in Rajasthan, India showed that among the initially tested IgM-negative patients, HEV RNA could be detected in the feces or serum of 13 patients. This finding indicated that low antibody titer may not reflect the viral load (6). Another study showed that the duration of viremia exceeded the time for transaminase to return to normal, which also indicated that liver damage may be unrelated to virus replication (15). Since the HEV itself is not associated with liver damage, it is likely that the body's own immune response causes liver damage as it clears the virus.

In order to prove that virus-induced host immune response is an important mechanism leading to hepatic cytopathic injury, many studies have explored the changes of the immunity in AHE patients through both *in vivo* and *in vitro* experiments. Immunohistochemical (IHC) staining of liver tissues from patients with acute liver failure (ALF) caused by HEV showed that the levels of CD3, CD8 (cytotoxic T cells), Granzyme B (granzyme B: Activated cytotoxic T cells and NK cell markers), CD56 (natural killer cells), CD4 (helper T cells), and CD8/CD3 and CD4/CD3 ratios were higher than those in normal liver tissue. IHC staining results showed that activated CD8^+^ T cells and NK cells were present in liver tissues infected with HEV (1). Analysis of the peripheral blood PBMC of patients with AHE showed that the number of NK and NKT cells was significantly lower than that of the healthy control group, but the number of activated NK and NKT cells was significantly higher than that of the healthy control group, and the number and activation status of NK and NKT cells returned to normal in the recovery period of AHE (17). By studying the cells of liver and peripheral blood of AHE, it was found that CD8^+^ T cells and their specific adaptive immune response play an important role in the pathogenesis of hepatitis E. In addition, NK cells and NKT cells play roles in the pathogenesis of acute HEV infection. NK and NKT cells constitute the main part of liver lymphocytes among which NK cells are large granule lymphocytes, accounting for 10–15% of peripheral blood mononuclear cells (18, 19), and 30% of intrahepatic lymphocytes. The interaction of perforin-granzyme and Fas-FasL can mediate cell death and activate other cells by secreting cytokines, thereby inhibiting virus replication. NKT cells are a kind of unconventional T cells that simultaneously express the cell surface characteristics of both NK cells (CD56) and T cells (CD3) (18). During the hepatotropic virus infection, the highly expressed CD1d on the surface of liver cells will cause the activation of NKT cells, and NKT cells can produce γ-interferon (IFN-γ) and IL-4 (15, 20). Comprehensive IHC study of the liver tissue and analysis of peripheral blood cells of AHE patients showed that the total number of NK cells and NKT cells in peripheral blood decreased, but its activation increased, while NK cells and NKT cells in the liver tissue increased, indicating that the increase of the number of cells illustrates the possible acute HEV infection, because the activated NK and NKT cells might migrate from peripheral blood to infiltrate the liver, and play a role in killing viruses in the liver (17).

In the study of hepatitis B virus (HBV) and hepatitis C virus (HCV) infection, it was found that cytotoxic CD8^+^ T cells mediate direct killing of hepatocytes, while CD4^+^ T cells inhibit virus replication and activates host macrophages by producing IFN-γ and tumor necrosis factor- α (TNF-α) and clear the virus with a non-cytopathic mechanism (21). In the absence of an effective HEV virus and cell co-culture system, it is difficult to determine whether the HEV virus directly affects the pathogenesis of host hepatocytes. Studies have shown that HEV particles are small, non-enveloped, 32–34 nm in diameter, and icosahedral symmetric. The viral gene is about 7.2 kb in length, is single-stranded, positive, and polyadenylated RNA containing three open reading frames (ORF): ORF1 encodes non-structural protein responsible for viral genome replication and multi-protein processing, while ORF2 encodes major capsid proteins. ORF3 encodes a phosphoprotein associated with the regulatory cytoskeleton (22). Normally, studies used HEV ORF2 protein (pORF2) and HEV ORF3 protein (pORF3) to stimulate patient-derived lymphocytes or transfect hepatoma cells, where ORF2 has been shown to have high immunogenicity and can effectively stimulate a specific T-cell response *in vitro* to protect primates from HEV infection (19). It was found that patients with AHE showed an acquired immune response to PORF2 and PORF3 in the form of a lymphoproliferative response. Peripheral blood mononuclear cells (PBMC) derived from patients with AHE were stimulated with pORF2 and pORF3 *in vitro*, and TNF-α^+^ CD4^+^ T cells and antigen-specific B cells that can produce IgG antibodies are significantly higher than healthy controls (21). Therefore, it is suggested that HEV is similar to HBV and HCV infection. After the onset of AHE, HEV-specific immune response occurred, and its level decreased with the decrease of anti-HEV lgM antibody titer and the normalization of liver function. Therefore, the immune response is considered to be a pathogenic factor in the development of hepatitis E, and also the main force to clear the virus (Table 1).

**Table 1:** Immune cells and cytokines in hepatitis E related diseases.

**References**	**Country**	**Immune cells and cytokines**	**Cases**
Prabhu et al. (1)	India	CD8^+^ T cells	37 patients with acute liver failure (ALF) because of HEV infection; Acute hepatitis A (*n* = 1); Acute hepatitis B (*n* = 6); Acute hepatitis C (*n* = 6);
Srivastava et al. (17)	India	NK and NKT; cells	41 patients with acute hepatitis E
Jilani et al. (23)	India	CD4 cells;	Pregnant FHF (*n* = 50);
		CD8 cells	Non-pregnant FHF (*n* = 50)
Moller et al. (24)	US	CD163^+^; macrophages	ALF (*n* = 100)
Wu et al. (12)	India	Mono-macs; dendritic cell;	AVH-E (*n* = 44);
		Macrophages	Pregnant females with AVH-E (*n* = 12);
			ALF-NE (*n* = 5)
Das et al. (25)	India	NK cells;	Patients in the acute phase of hepatitis E infection (*n* = 86);
		IFN-γ;	HEV recovered individuals (*n* = 101)
		TNFα	

## Distribution of Immune Cells and Related Cytokines in HEV-ACLF Patients

Using IHC analysis of lymphocyte subsets in liver tissue infiltration and distribution, a study found that patients with ACLF had increased intrahepatic CD4^+^ T cells, CD8^+^ T cells, and NK cells compared with the chronic hepatitis and normal control group. Meanwhile, the study also showed that patients with ACLF had intrahepatic lymphocytes count about 50 times more than normal subjects. This finding indicated that the high concentration of intrahepatic lymphocytes in ACLF patients compared with normal people is caused by the infiltration of peripheral blood lymphocytes into the liver (1, 17). The low circulating lymphocytes in the peripheral blood of ACLF patients increases opportunistic infection and cause endotoxemia, and may activate monocytes and macrophages to release high levels of TNF-α, thereby aggravating the inflammatory damage of liver tissue (26, 27). The activation of EM cells is related to the increased expression of transcription factor T-Bet, and the activation of EM cells is mediated by IFN-γ after stimulation, the level of T-Bet increased further. The expression of T-Bet is related to the increased levels of chemokines including CXCR3, Granzyme B and CD122, which can drive EM cells to migrate from peripheral blood to liver (28). In addition to the above cytokines, IL-15, IL-18, CXCL8, CXCL9, CXCL10, and CCL2 promote the chemotaxis of immune cells to liver tissue, among which CXCL8 and CCL2 can promote the recruitment of non-adaptive immune cells such as monocytes to liver (29). The infiltration of lymphocytes into liver tissue is a multi-step process mediated by the interaction between multiple adhesion molecules, chemokines and chemokine receptors released by immune cells such as lymphocytes, monocytes and macrophages (30). Since the infiltration of CD4^+^ T cells and CD8^+^ T cells from peripheral blood into liver tissue was also observed in acute hepatitis E and acute liver failure during pregnancy, it was inferred that the infiltration of CD4^+^ T cells and CD8^+^ T cells in acute and chronic hepatitis E caused by HEV might cause ALF (1, 23).

The study for the gene expression profile of liver biopsy in patients with HEV liver failure showed that compared with normal liver tissue, 1,703 genes were up-regulated and 1,674 genes were down regulated in liver tissue (31). Among them, 1,235 differential genes were related to immune system pathway, mainly related to cellular immune mechanism. such as T-cell toxic surface molecules, CTL mediated immune response, T helper cell surface molecules, costimulatory signaling in T cell activation pathway, T cell receptor signaling pathway, TCR activation signaling pathway initiated by Lck and Fyn tyrosine kinase, T cell receptor and CD3 complex signaling pathway, and IL-17 signaling pathway. qRT-PCR also showed that the mRNA levels of these genes were in good agreement with the microarray results, and the surface molecules of T cells such as Fyn and NF were found κB1, protein kinase C, and CTLA4 are over expressed in liver tissue, which indicated that activated CD8^+^ T cells play a role in liver injury during HEV infection.

Acute and chronic hepatitis caused by hepatitis E virus and acute liver failure are also caused by the infiltration of CD4^+^ T cells and CD8^+^ T cells (32). In fact, the transport of lymphocytes to the liver is a major immune monitoring mechanism. Intrahepatic lymphocytes are essential for virus clearance or control of viral infection. However, continuous transport of lymphocytes to the liver may lead to extensive necrosis of hepatocytes and eventually lead to liver failure.

## Intrahepatic Immune Microenvironment in HEV-ACLF Patients

IHC showed that there were more CD4^+^ T cells in the liver of AHE patients than in the healthy control group. The increase of CD4^+^ T cells, from the peripheral blood mononuclear cell count to the IHC analysis of the cell infiltration in the liver, was observed in patients with acute and chronic hepatitis E-induced liver failure. The amplification of CD4^+^ T cells that produce IFN-γ and TNF-α play important roles in limiting HEV replication and resolving HEV infection (1). CD4^+^ T cells produce cytokines that promote the differentiation of CD8^+^ T cells into cytotoxic T cells (CTL), which can directly kill infected liver cells and promote liver cell damage while helping to clear the virus. Data from liver biopsies indicate that similar infiltration of CD8^+^ T cells can be seen in liver tissues of ALF caused by hepatitis E and other hepatitis virus infections, and the data indicate that CD8^+^ cell population exceeds CD4^+^ cells (1). Therefore, CD8^+^ T cell-mediated cellular immunity, which can directly kill the hepatitis virus, plays a key role in the progression of hepatitis E.

**Figure 1:**
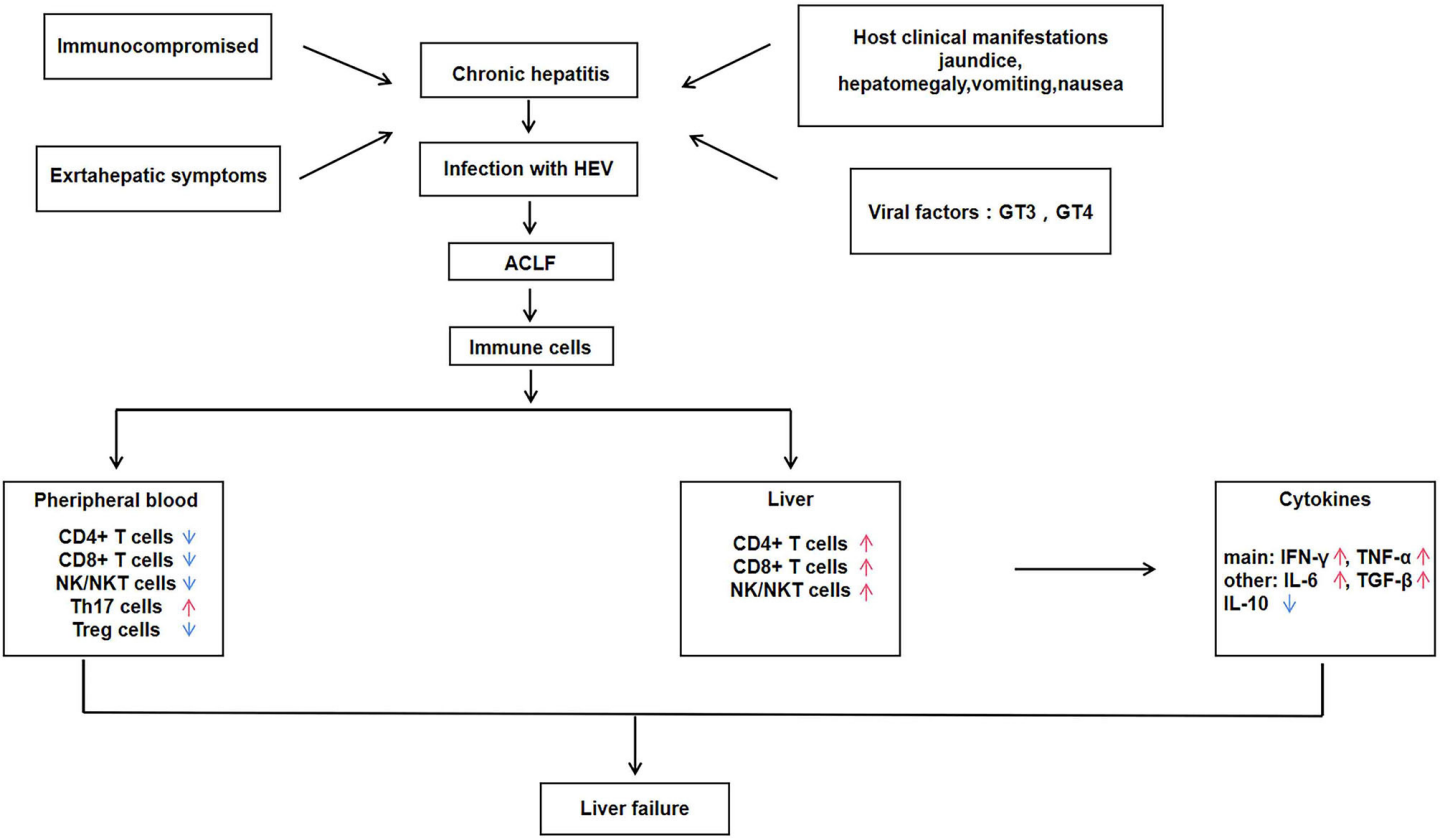
Immune regulatory mechanisms of hepatitis E associated acute and chronic liver failure (HEV-ACLF).

It is well-known that adaptive immune response includes antigen presentation, activation of lymphocytes, formation of immune molecules and occurrence of immunological effects (Figure 1). In the stage of antigen recognition, T lymphocytes and B lymphocytes can accurately recognize antigen through TCR and BCR, respectively, while T lymphocytes can only complete subsequent activation and proliferation and kill target cells after antigen presentation by antigen presenting cells. Some studies have discussed the influence and mechanism of monocytes and macrophages in the pathogenesis of AHE. Previous studies on patients and chimpanzees infected with hepatitis E virus have shown that the presence and role of inflammatory macrophages in acetaminophen-induced ALF have been elucidated and understood (32). In addition, in the subsequent model of carbon tetrachloride induced liver injury, the number of CD11b^+^ F4/80^+^ or F4/80^−^ or CD11b^+^ CD163^+^ inflammatory macrophages was significantly increased, and the infiltration of macrophages in liver tissue led to severe liver injury (24, 33–35). Monocytes and macrophages recognize pathogens through members of the Toll receptor family TLR3 and TLR8, and then secrete reactive oxygen species (ROS) to mediate phagocytosis (12). The TNF-α secreting macrophages amplified from circulating monocytes are known as activated inflammatory M1/M2 macrophages. Activated macrophages can produce enough ROS to kill pathogens and resolve infection, while its excessive production of TNF-α leads to liver inflammation, injury, and liver functional failure (36). In addition, the activation of macrophages can also produce IFN-α, IL-12, and IL-18, which can further trigger the inflammatory immune response and lead to tissue damage (37). However, the study found that although the number of macrophages in AHE patients was significantly increased compared to the healthy control individuals, the production of ROS in macrophages was low, indicating that the impaired function of macrophages led to its dysfunction and inactivation (12). The phenomenon that the number of monocytes increases while their function is impaired is caused by the secretion of TNF-α and inhibitory cytokines such as IL-10 and TGF-β by macrophages (12). A study on the damage of TLR signaling pathway during HEV infection has explained the impairment of monocyte and macrophage function in patients with acute liver failure infected with hepatitis E. Studies have shown that macrophage function activation and HEV clearance are caused by TLR3 signaling activation of MyD88-independent pathway and TLR9 signaling activation of MyD88-dependent pathway through TRIF, TRAF3, and TRAF6. However, the expression of TLR3 and 7 was reduced in AHE patients, and the MyD88-dependent pathway activated by TLR9 signaling and the downstream signaling of TLR were damaged, which resulted in reduced ROS release and impaired phagocytosis of monocytes and macrophages as well as the release of cytokines such as TNF-α and IFN-γ, leading to inefficient treatment of HEV pathogens (12).

NK cells play a crucial part in the initial response to viral infection and other pathogens is a vital component of the immune system (38). In the process of defense against viral infection, NK cells induce production of IFN-α/β and other natural cytokines, thereby inhibiting viral replication and enhancing cytotoxicity to target cells (39). A large number of studies have shown that immune-activated CD8^+^ T cells, NK cells, and NKT cells in peripheral blood and liver can induce severe liver injury in patients infected with HEV (26, 27). NK cells are momentous in the recognition and initial response of HEV. In the battle against HEV infection, NK cells are believed to be the first attack barrier formed and can call on T and B lymphocytes, that in turn induce an adaptive immune response (40). NKT cells represent a small number of lymphocytes and exhibit characteristics of both T cells and NK cells (41). The function of NK cells is integrated and controlled by signals from various activation and inhibition receptors, that bind to pathogens and activate NK cells (42). Among them, the most effective NK cell activated receptors (NAR) are ADCC-mediated molecules, including CD16, NKG2D, NKP30, NKP44, and NKP46. NKT cells expressing NAR exhibit highly specialized effector memory phenotypes (43, 44). NK and NKT cells expressing NARs account for 13% of peripheral blood lymphocytes and 50% of liver lymphocytes. The distribution, activation, cytotoxicity and effector function of NK/NKT cells were studied in 86 patients with acute exacerbation of HEV infection and 54 healthy controls during the development of the disease. It was shown that the percentage of NK (CD56^+^CD3^−^) and NKT (CD56^+^CD3^+^) cells in peripheral blood of AHE patients is lower than that of healthy control individuals, while the NK and NKT cells expressing NARS (NKP44 and NKP46) are more than that of healthy control individuals (25). The number of NK and NKT cells was significantly increased in an IHC study of liver biopsies from HEV-infected patients with acute liver failure. These results demonstrate that activated NK/NKT cells migrate from the peripheral blood and infiltrate into the liver to fight against virus during acute or chronic HEV infection. IFN-γ is produced by various types of cells, including monocytes, dendritic cells, NK cells, and NKT cells, with NK and NKT cells as the main sources of IFN-γ in the liver. Therefore, the above studies on various immune cells during the pathogenesis of ACLF suggested that IFN-γ produced by cytotoxic CD8^+^ T cells and CD4^+^ T cells in the liver can recruit macrophages, NK cells and NKT cells during the pathogenesis of AHE patients.

**Figure 2:**
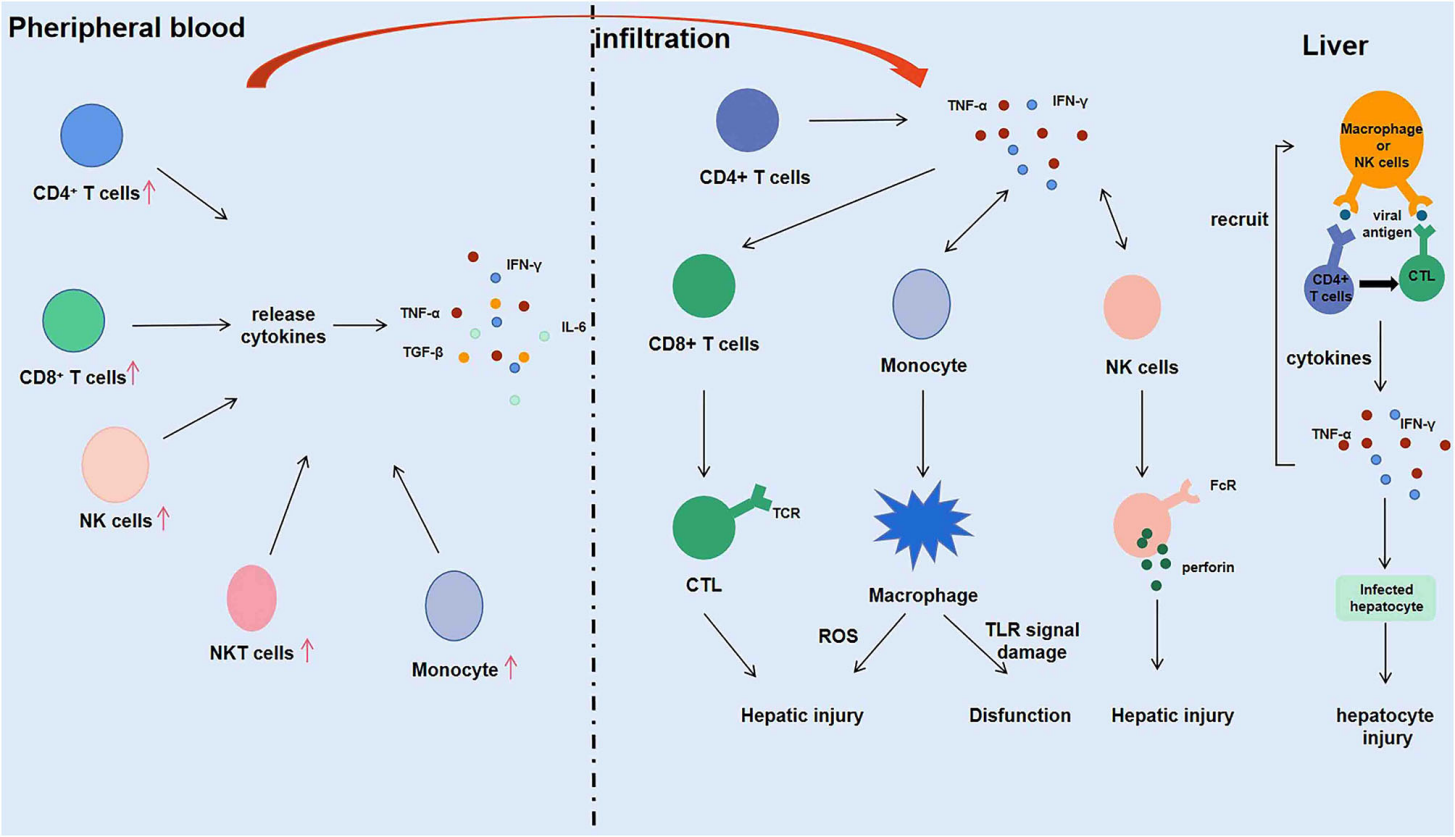
Intrahepatic immune microenvironment in hepatitis E associated acute and chronic liver failure (HEV-ACLF) patients.

Macrophages infiltrating into the liver mediate phagocytosis by releasing ROS, and NK cells play a killing role by secreting perforin. FcR on the surface of macrophages and NK cells binds to the Fc segment of antibodies binding to virus-infected cells, mediating killing of virus-infected liver cells (17). Firstly, macrophages and NK/NKT cells initially recognize and kill HEV and HEV-infected liver cells through primary immune response. Then, macrophages and NK cells present viral antigens to T and B lymphocytes in the liver. After recognizing the antigen, the lymphocytes are activated, proliferated, and differentiated to produce effector cells (such as CTL) and effector molecules (such as IFN-γ, which is the most important one in the process of HEV infection). Finally, the effector cells and effector molecules are used to eliminate the hepatitis E virus and the virus-infected hepatocytes. IFN-γ secreted by immune cells during the immune response further activates and recruits macrophages, NK cells, and NKT cells, promoting differentiation of CD8^+^ T cells into CTL, which kills infected liver cells and promotes liver damage while helping to clear HEV. Conversely, the immune response is weakened in organ transplant patients with immunosuppressive treatment, which might lead to a decrease in CD8^+^ T cells, CD4^+^ T cells, and the cytokines they produced. Although it protects the liver from damage, it also causes the hepatitis E virus to be unable to be removed from the body, making hepatitis E chronic. In conclusion, the immune microenvironment of the liver plays an essential role in the development of hepatitis E virus (Figure 2).

## Distribution of Other Immune Cells in HEV-ACLF Patients

In addition to the above-mentioned immune cells that play a key role in the pathogenesis of HEV-ACLF, studies have shown that regulatory T cells (Treg cells) and IL-17-producing helper T cells (Th17 cells) also play a crucial role in the immune mechanism of HEV-ACLF (45). Treg cells mainly play the function of immunosuppression. Treg cells accumulate and expand in the infected site to play the immunosuppressive activity (46), and the deficiency or destruction of Treg cells will lead to autoimmune and inflammatory diseases in human and animals (47). Th17 cells are involved in the development of inflammation and autoimmune diseases by stimulating the production of a large number of inflammatory chemokines and inflammatory cytokines, and promoting the recruitment of neutrophils in tissues (48). The changes in the level of IFN-γ is similar to the trends of Th17 cells in the pathogenesis of HEV-ACLF, and high level of IFN-γ is involved in severe liver injury in patients with acute liver failure (49). The proportion of Th17 cells in peripheral blood was significantly increased in HEV-ACLF patients, suggesting that Th17 cells and IFN-γ are involved in promoting and maintaining inflammatory response and aggravating liver injury in HEV-ACLF patients. The number of Treg cell in HEV-ACLF patients showed a continuous decline and then stabilized, and the concentration of cytokine IL-10 was consistent with the dynamic changes of Treg cells. It is known that the increase of Treg cells and cytokines can inhibit the T cell response to regulate liver tissue inflammation, and the increase in their proportion may be a feedback to the increase of Th17 cells and pro-inflammatory cytokines, and suppress the immune response in the process of chronic liver failure to achieve a balance between pro-inflammatory and anti-inflammatory immunity (45).

In the pathogenesis of HEV-ACLF, pro-inflammatory immunity was significantly up-regulated, while anti-inflammatory immunity was significantly down-regulated, and the proportion of Th17 cells in peripheral blood was significantly increased. However, the number of Treg cells was decreased instead of increased, suggesting that there may be defects in the function of Treg cells. Therefore, the imbalance of Treg/Th17 is an important cause of liver function injury in patients with acute or chronic liver failure (45). The roles of T lymphocytes, regulatory T cells and helper T cells in the process of liver injury are closely related to the cytokines that they produce. It has been shown that IFN-γ and TNF-α in the liver are significantly associated with infiltration of aggregated CD4^+^ and CD8^+^ T cells in the liver, and that the expression of IFN-γ is unique to lymphocytes, whereas TNF-α is mainly produced by intrahepatic macrophages (12). The expression of IFN-γ and TNF-α in the liver of HEV-ACLF patients was significantly higher than that of normal controls, while the expression of anti-inflammatory cytokine IL-10 was not different between HEV-ACLF patients and normal controls.

Therefore, the upregulation of IFN-γ and TNF-α in the liver of HEV-ACLF patients is not offset by IL-10, and the imbalance of the expressions of pro-inflammatory cytokines and anti-inflammatory cytokines in the liver of patients is the important immune mechanism of HEV-ACLF liver injury. At the same time, the regulation of pro-inflammatory liver environment by cytokines may provide new ideas and strategies for the prevention and treatment of HEV-ACLF.

## Treatment of HEV-ACLF Patients

In general, for patients with AHE with normal immunity, only symptomatic treatment is usually required because the duration of viremia is short. HEV infection can lead to chronic hepatitis in organ transplant patients who are treated with immunosuppressive agents. Studies have shown that about 30% of patients can clear the virus spontaneously by reducing immunosuppressive therapy. Clinically antiviral therapy is usually used when this approach is not successful, and the one currently used with the highest potency, efficacy and safety is ribavirin (50). Ribavirin is an effective antiviral drug that inhibits the synthesis of viral mRNA by inhibiting a variety of RNA and DNA, thus preventing viral replication (51).

Ribavirin is used to treat bronchiolitis and pneumonia caused by respiratory syncytial virus in infants and young children, chronic HCV, as well as a variety of viral infections such as viral upper respiratory tract infections. And ribavirin can be used to treat AHE and reduce the severity of the disease in patients with acute and chronic liver failure. It has shown good efficacy in the treatment of patients with chronic HEV infection, including patients with AIDS and leukemia with chronic hepatitis E, as well as transplant patients with chronic HEV infection and patients with rheumatic disease with immunosuppression. Therefore, ribavirin is the drug of choice for the treatment of HEV infection in most cases. Studies have shown that a course of 3 months is the optimal duration of ribavirin monotherapy, with longer treatment periods available for patients with severely compromised immune function. Prospective studies are needed to determine the optimal duration of ribavirin and the most beneficial therapeutic dose (52).

Although ribavirin is the preferred drug for the treatment of hepatitis E virus infection, there are many side effects of its single drug treatment. The most significant ones are severe anemia, the reduplication of the virus and the recurrence of the disease after discontinuation of treatment, and fetal deformity, Kamar et al. (50). A large number of studies have also provided solutions to the above side effects. For example, for patients with severe anemia caused by ribavirin treatment, combination therapy with another HEV inhibitor or direct acting antiviral drugs can be considered to reduce the amount of ribavirin (52). In addition, it was found that treatment with ribavirin was associated with increased heterogeneity in the open reading frame of the virus. Virus sequencing of the sera of a few patients who had failed treatment with ribavirin revealed the presence of G1634R mutation in viral polymerase. *In vitro* studies showed that G1634R mutation increased HEV replication capacity (53). Recent data suggest that mutations in G1634R, including K1382N, Y1587F, D1384G, V1479I, and K1398R, are present in patients who relapsed during ribavirin therapy, and all of these mutations increase the replication capacity of hepatitis E virus, and increase the antiviral activity of ribavirin. Although there is currently no alternative treatment for ribavirin, *in vitro* studies have shown that the combination of sofebuvir and ribavirin may inhibit HEV replication and increase the antiviral effect of ribavirin. Sofebuvir is a nucleotide polymerase inhibitor of HCV infection that blocks HEV replication *in vitro* by inhibiting viral RNA-dependent RNA polymerase. Some patients treated with sofebuvir may develop liver fibrosis, cirrhosis, and liver failure, so the potential effect of *in vivo* two-drug combination on HEV replication needs to be determined (53).

In addition, ribavirin is contraindicated for pregnant women because of the medication-induced fetal deformities, however, untreated HEV poses a high risk to both mother and fetus. Drug trials may therefore be helpful in these patients, and acute hepatitis E often occurs in the third trimester, when fetal organ development is complete. Previous research data did not observe a clear teratogenic effect of ribavirin in humans. Nevertheless, further research on ribavirin treatment of AHE infection during pregnancy is needed (54).

Besides ribavirin, polyethylene glycol interferon alpha (PEG-IFN-α) is another drug that can be used for hepatitis E. The mechanism of action of PEG-IFN-α is that interferon binds to the specific alpha receptor on cell surface, triggers the complex signal transduction pathway in the cell and activates gene transcription, thereby regulating a variety of biological effects, including inhibition of replication of viruses in infected cells, inhibiting cell proliferation, and playing an immune regulatory role. Studies have shown that PEG-IFN-α can be used for liver transplant recipients infected with HEV, but other solid organ transplant patients such as heart transplant, lung transplant and kidney transplant are not recommended because interferon has immune-stimulating effects and can increase the risk of acute immune rejection in organ transplant patients (55).

In addition, interferon activates the body's immune system, removing the virus while damaging infected liver cells. Moreover, similar to ribavirin, PEG-IFN-α may cause fetal deformities, so it should not be used in pregnant women. Studies *in vitro* have observed a slight synergistic effect of ribavirin and interferon-α combination, which can reduce the dosage of ribavirin and potentially avoid anemia and other side effects. This finding suggested that the combination regimen can be use in the clinical setting for the treatment of hepatitis E (56). Based upon, it can be seen that safe and effective treatment of hepatitis E is urgently needed. In the case of acute HEV infection, the use of effective antiviral drugs to shorten the course of the disease can prevent the disease from progressing to fulminant liver failure and effectively prevent transmission of the virus during epidemics and outbreaks. As a high-risk group of hepatitis E, pregnant women have no therapeutic drugs and methods at present. When studying therapeutic drugs, teratogenicity and major adverse consequences should be avoided as far as possible to save the lives of pregnant women and fetuses. By the above content, the changes in hormone levels in pregnant women is one of the factors that lead to HEV infection. Therefore, modulating the hormonal system may be an effective treatment option. High concentrations of progesterone have been shown to inhibit CTV replication. Supplementation of progesterone in pregnant women with hepatitis E may be considered, and more suitable models *in vitro* and *in vivo* need to be developed for antiviral studies (57).

At present, the side effects and limitations of available therapeutic drugs promote the development of new antiviral drugs, and explore novel therapeutic targets from the perspectives of both the virus and the host. Ribavirin and PEG-IFN-α affect virus and host, respectively. Ribavirin inhibits viral replication by inhibiting the synthesis of viral mRNA. PEG-IFN-α regulates a variety of biological and immunological effects of the host and plays a role in inhibiting virus replication by binding to the specific receptors on cell surface. Antiviral drugs include inhibitors that inhibit virus entry into the host body, RNA-dependent RNA polymerase inhibitors, viral methyltransferase inhibitors, HEV helicase inhibitors, and inhibitors targeting other viral proteins (58). There are studies suggested that HEV replication may be affected by sex hormones, for example, the level of progesterone in pregnant patients may cause adverse outcomes. Some studies have found that HEV infection mainly affects men over 50 years of age in developed countries, that low testosterone may promote HEV replication, and that testosterone supplementation may be beneficial for older male patients with chronic HEV infection. Similarly, drugs that affect estrogen such as estrogen receptor modulators (tamoxifen and raloxifene) may improve the treatment of menopausal women with chronic HEV infection (59). Therefore, modulating the hormonal system might be an alternative approach to the treatment of hepatitis E. However, there is no specific treatment strategy on this approach, which requires extensive study.

## Conclusions and Perspectives

Hepatitis E is an infectious disease not only affects the underdeveloped countries, but also threads the economically developed countries. The two types of intestinal hepatitis, hepatitis E and hepatitis A, are very similar in terms of acute infection time and clinical features. Therefore, the early understanding and study on hepatitis E was based on models of hepatitis A. However, with the development of technology and in-depth research on hepatitis E, it was known that hepatitis E has similarities with hepatitis A and other types of viral hepatitis, as well as its unique characteristics. This article discusses the immunological manifestations and immune regulatory mechanisms of hepatitis E and HEV-ACLF. It is obvious that the immunological response is very important for the development and progression of hepatitis E, and the immune environment in the liver is very complicated. In addition to the immune cells (T lymphocytes, regulatory T cells, NK cells, and macrophages) and immune molecules (IFN-γ and TNF-α) mentioned in the article, there may be other immune cells and immune molecules that may play important roles.

Currently, the protective measures and treatment methods for patients with AHE and HEV-ACLF are very limited. It is necessary to develop HEV-specific cell models and animal models, and establish three dimensional organoid liver cell culture system for further study on the immunological mechanism of hepatitis E. Such studies will be valuable for the eradication of hepatitis E.

## Author Contributions

JW and YG had the idea for the article. BL and GZ performed the literature search and data analysis. NG and JW drafted and critically revised the work. ML revised the draft. All authors contributed to the article and approved the submitted version.

## Conflict of Interest

The authors declare that the research was conducted in the absence of any commercial or financial relationships that could be construed as a potential conflict of interest.

## Publisher's Note

All claims expressed in this article are solely those of the authors and do not necessarily represent those of their affiliated organizations, or those of the publisher, the editors and the reviewers. Any product that may be evaluated in this article, or claim that may be made by its manufacturer, is not guaranteed or endorsed by the publisher.
